# Thymol and Limonene as Potent Antimicrobials: Bridging In Vitro Efficacy and Molecular Docking Insights

**DOI:** 10.1155/ijm/2768549

**Published:** 2026-09-10

**Authors:** Nesrine Benkhaira, Mohamed El Fadili, Saad Ibnsouda Koraichi, Kawtar Fikri-Benbrahim

**Affiliations:** ^1^ Department of Biology, Faculty of Sciences and Technologies, Laboratory of Microbial Biotechnology and Bioactive Molecules, Sidi Mohamed Ben Abdellah University, Fez, Morocco, usmba.ac.ma; ^2^ Department of Chemistry, Faculty of Sciences Dhar El Mehraz, Laboratory of Engineering, Modeling, and Analysis of Systems (LIMAS), Sidi Mohammed Ben Abdellah University, Fez, Morocco, usmba.ac.ma

**Keywords:** ADMET, docking, fungi, in silico, in vitro, limonene, *Mycobacterium*, thymol

## Abstract

Nowadays, antimicrobial resistance has created an urgent need for elaborating effective and novel antibiotic agents. In effect, bioactive compounds derived from plants have attracted great interest due to their biological activities. This work intended to study and compare the antimicrobial effect of thymol and limonene against multiple clinical microorganisms including gram‐positive bacteria, gram‐negative bacteria, yeasts, and mycobacteria. Molecular docking and bioavailability prediction of thymol and limonene were also performed. The in vitro study was realized via disk diffusion technique, minimum inhibitory concentration (MIC), minimum bactericidal concentration (MBC), and minimum fungicidal concentration (MFC) assays. In parallel, in silico study of these compounds evaluated their drug‐likeness, pharmacokinetic, and toxicity parameters, and binding to bacterial targets. Findings showed that thymol exhibited significantly greater antimicrobial efficacy than limonene against all microorganisms. The compounds demonstrated potent antimicrobial activity against bacteria (12.0 ± 1.7–33.1 ± 2.1 mm), yeasts (15.87 ± 0.81–24.5 ± 0.8 mm), and mycobacteria (8.5 ± 0.5–18.4 ± 2.1 mm). These findings were supported by low MIC and MBC/MFC (8–128 *μ*g/mL), whereas MBC/MIC and MFC/MIC ratios (< 4) confirmed their bactericidal and fungicidal effect. Thymol also exhibited higher antimicrobial effect than the reference antibiotics (kanamycin, rifampicin, fluconazole, and chloramphenicol) against the tested microorganisms Moreover, in silico data showed that these compounds satisfy the main standards for drug‐like molecules, with simulations pointing to good oral absorption, an acceptable safety profile, and stable binding to key enzymes in the studied microorganisms, which supports their antibacterial potential. Overall, these combined experimental and computational results point to thymol and limonene as promising natural antimicrobial agents.

## 1. Introduction

The global epidemiological spread of antimicrobial resistance (AMR) constitutes one of the most serious public health of the 21st century [1]. The AMR is responsible for an estimated 1.27 million deaths annually worldwide, with nearly 4.95 million deaths associated with resistant bacterial infections [2].

Over the past years, the excessive usage and suboptimal application of standard antibiotics have led to the development of multidrug‐resistant pathogens [3]. A well‐known example is the misuse of methicillin, which has led to the emergence of a major nosocomial pathogen called methicillin‐resistant *Staphylococcus aureus* (MRSA) [4]. Furthermore, the overuse of fluoroquinolones has also contributed to the emergence of antibiotic‐resistant strains of *Escherichia coli*, reducing treatment options for common urinary infections [5].

In response to the reduced effectiveness of conventional antibiotics, scientific research is largely focusing on natural substances as promising alternative sources of antimicrobial agents [6]. Indeed, medicinal and aromatic plants, particularly their essential oils (EOs), have been mainly examined for their potential biological properties. More recently, researchers have focused on active constituents responsible for these properties, especially monoterpenes which include major bioactive molecules such as thymol and limonene [7].

Thymol (C_10_H_14_O) is one of the naturally occurring monoterpenoids that falls under the class of phenols. It is also named 2‐isopropyl‐5‐methylphenol and differs from the compound carvacrol by being an isomer with the same molecular formula [8]. Thymol is found in many parts of EOs from different medicinal plants and mostly belongs to the taxonomic families Lamiaceae and Apiaceae, such as *Thymus* and *Origanum* and other aromatic plants [9]. Thymol has attracted considerable attention for its important diverse biological properties, especially wound‐healing, antispasmodic, antioxidant, anti‐inflammatory, and anticancer effects [10–12].

Limonene (C_10_H_16_), also called 1‐methyl‐4‐isopropenylcyclohex‐1‐ene or dipentene, is a naturally occurring monocyclic monoterpene that can exist as a pair of optical isomers with D‐limonene being the most common isomer present in nature [13]. It occurs primarily in the EOs of citrus fruits and can comprise over 80% of the total oil extracted from the fruit [14]. In addition to its usage in the food and cosmetic industries, there has been considerable scientific interest around limonene due to its many biological activities including antimicrobial, antioxidant, anti‐inflammatory, analgesic, and neuroprotective activities [15].

Previous studies have shown that thymol and limonene exhibit strong antimicrobial potential [16]. Thymol‐rich EOs, in particular from *Origanum* and *Thymus* species, demonstrated strong antioxidant activity as well as antibacterial activities against clinically significant pathogens such as *Streptococcus iniae* [17]. Similarly, limonene registered bacteriostatic and bactericidal effects against both gram‐positive and gram‐negative bacteria, including resistant *Enterococcus faecium* [18].

Although limonene and thymol have been widely investigated for their antimicrobial properties, comparisons between these monoterpenes are generally indirect because they originate from independent studies using different microbial strains, experimental methods, and evaluation protocols. Sch variability makes it difficult to accurately assess their relative antimicrobial potential.

To the best of our knowledge, no previous study has directly compared thymol and limonene under identical experimental conditions while integrating molecular docking analyses to explore their interactions with microbial target proteins. Therefore, the present work was designed to provide a comprehensive side‐by‐side evaluation on the antimicrobial efficacy, bactericidal/fungicidal activity, and molecular interactions of these two bioactive monoterpenes using standardized experimental and computational approaches.

## 2. Material and Methods

### 2.1. Microorganisms

The present work evaluated the antibacterial properties of thymol and limonene against three gram‐negative bacteria, *Pseudomonas aeruginosa* 034 (clinical isolate), *E*. *coli* ATCC 25922, and *Salmonella enterica* (clinical isolate), and three gram‐positive bacteria, *Staphylococcus aureus* 017 (clinical isolate), *Bacillus subtilis* ATCC 6633, and *Micrococcus luteus* ATCC 14452. Furthermore, this study examined the effect of the two compounds against mycobacterial strains, *Mycobacterium aurum* A+ and *Mycobacterium smegmatis* mc^2^155, as well as two clinical yeast isolates, *Candida albicans* and *Candida tropicalis*. All microbial strains were maintained under appropriate culture conditions before any manipulation.

### 2.2. Tested Compounds and Reference Antibiotics

Thymol and limonene, with a purity of ≥ 98%, were obtained from Sigma‐Aldrich (St. Louis, Missouri, United States) and selected as the bioactive compounds evaluated in this study. Stock solutions were prepared at 10 mg/mL and used in the antimicrobial assays. To validate the antimicrobial assays, standard antibiotics and antifungal agents were used as positive controls, namely ampicillin (AMP) (10 *μ*g/disk), chloramphenicol (CHL) (30 *μ*g/disk), fluconazole (FLC) (25 *μ*g/disk), and rifampicin (RIF) (5 *μ*g/disk), following the recommendation of Clinical and Laboratory Standards Institute (CLSI) guidelines [19].

### 2.3. Agar Disk Diffusion Assay

Antimicrobial activity was determined using the disk diffusion method. Briefly, bacterial and fungal suspensions were prepared and adjusted to a turbidity of 0.5 McFarland. Mueller–Hinton (MH) agar plates (for bacteria), Sauton agar (for mycobacteria), and Sabouraud dextrose agar plates (for yeasts) were inoculated by the microbial suspension. Sterile filter paper disks (6 mm in diameter) were impregnated with 10 *μ*L of thymol or limonene solution (10 mg/mL), that is, 100 *μ*g per disk, and then placed on the surface of the inoculated agar. Standard antibiotic disks were also placed on the same plates for comparison. The plates were incubated at 37°C for 18–24 h for bacteria and 48 h for yeasts. Slow‐growing organisms were incubated under suitable conditions. After incubation, three independent experiments were performed to determine the diameter of the inhibition zones (mm) [20]. The results are expressed as mean ± standard deviation (SD).

### 2.4. Minimum Inhibitory Concentration (MIC) Determination

The MIC values of thymol, limonene, and reference antibiotics were determined by the broth microdilution method in sterile 96‐well microplates [20]. Briefly, appropriate culture media were used to prepare two‐fold serial dilutions of each compound, yielding final concentrations ranging from 4 to 256 *μ*g/mL. All wells were inoculated with standardized microbial suspensions adjusted to 0.5 McFarland. The plates were incubated under conditions appropriate for the type of microorganism (e.g., 24 h at 37°C for bacteria, 72 h at 35°C for mycobacteria, or 48 h at 30°C for yeasts). Positive control (culture medium and microbial suspension) and negative control (culture medium) wells were included to verify microbial growth and sterility. The microplates were incubated at appropriate conditions depending on the microorganism. Following incubation, microbial growth was evaluated by adding 10 *μ*L of resazurin, used as a redox‐based viability indicator. The MIC was defined as the lowest concentration of the compound resulting in complete inhibition of visible microbial growth.

### 2.5. Minimum Bactericidal Concentration (MBC) Determination

To determine the bactericidal or bacteriostatic nature of thymol and limonene, the MBC values were determined after detecting the MIC values. Indeed, 10‐*μ*L aliquots from wells showing no visible growth were subcultured onto fresh agar plates without any antimicrobial agent. The plates were incubated under appropriate conditions for the tested microorganism. The MBC value was defined as the lowest concentration of the compound that resulted in no visible bacterial growth on the agar plates after incubation, showing complete microbial inhibition. The MBC/MIC ratio was determined to identify the antimicrobial mode of action. A ratio less than or equal to 4 indicates bactericidal activity, whereas a ratio greater than 4 indicates bacteriostatic activity [21].

## 3. Physicochemical Properties, In Silico Prediction of Absorption, Distribution, Metabolism, Excretion, and Toxicity (ADMET) Parameters, and Bioavailability Radar

By carrying out an in silico analysis, the physicochemical and pharmacokinetic aspects of limonene and thymol as two of the primary constituents will be compared and contrastive. To initiate this process, the molecular structure will be obtained and transformed into the standard format needed to carry out computational analysis. The primary physicochemical descriptors to be calculated include molecular weight (MW), lipophilicity (Log *P*), topological polar surface area (TPSA), number of hydrogen bond donors (HBDs), number of hydrogen bond acceptors (HBA), rotatable bonds (RB), and molar refractivity (MR) [22–24]. The SwissADME web portal was used to predict ADMET profiles for limonene and thymol; this included assessing how soluble limonene and thymol would be in the gastrointestinal track, whether either compound would cross into the brain (blood–brain barrier [BBB]), potential interactions with cytochrome P450 (CYP) enzymes, and whether either compound would transfer readily through human skin. Drug‐like characteristics were determined through Lipinski′s rule of five (the rule states that if a drug has an MW < 500 Da; Log *P* < 5; less than five separate hydrogen‐bond donors; and less than 10 hydrogen‐bond acceptors, then it is likely to be drug‐like) [25]. Examples of other commonly used filters include types A & B of the pharmaceutical industry; lastly, medicinal chemistry friendliness will be assessed in terms of how well it passes many of the filters described above and how well it passes/does not pass as drug‐like using these same filters [26, 27].

To investigate oral bioavailability, we used the BOILED‐Egg model (Brain Or Intestinal EstimateD permeation method). This graphical method predicts the passive intestinal absorption and ability of a substance to cross the BBB based on the lipophilicity and polarity of the compound being tested [28–31]. Drugs that fall into the white section of the egg are expected to have good GI absorption and drugs that fall into the yellow yolk of the egg will have suitable passage across the BBB. We also generated a bioavailability radar to graphically represent the six physicochemical properties that are important in assessment of oral bioavailability; these are lipophilicity, size, polarity, solubility, flexibility and saturation. We used the generated radar plots to evaluate how drug‐like limonene and thymol will perform regarding their overall drug‐like properties and pharmacokinetic properties.

## 4. Molecular Docking

The computational studies using molecular docking were performed via the calculation of potential antimicrobial mechanisms for each of the two primary phytochemicals, limonene and thymol, against 10 different types of pathogenic microorganisms. Each compound was separately docked to each of the target protein structures to compute binding affinities and molecular interaction results with various antimicrobial targets that are biologically important. 3D crystal structures of the target proteins were obtained from the Protein Data Bank (PDB) [32]. The proteins of interest were selected due to their experimentally proven functions in the survival, pathogenicity, or metabolic pathways of corresponding pathogens, qualifying them as already identified antimicrobial targets. Furthermore, only those high‐resolution crystal structures with cocrystallized ligands included were considered in order to provide strict validation of the docking process in redocking before docking with limonene and thymol. The protein target families that were chosen corresponded to the 10 pathogenic microorganism families that were used in this study; *P. aeruginosa* 034 (clinical isolate from human; PDB ID: 2UV0), *E*. *coli* ATCC 25922 (Type Strain; PDB ID: 6F86), *S*. *enterica* (PDB ID: 5ZXM), *S. aureus* 017 (clinical isolate from human; PDB ID: 3G75), *B*. *subtilis* ATCC 6633 (Type Strain; PDB ID: 7W9Y), *M*. *luteus* ATCC 14452 (Type Strain; PDB ID: 3AQC), *M*. *aurum* A+ (PDB ID: 2X22), *M. smegmatis* mc^2^155 (PDB ID: 2X22), *C*. *albicans* (PDB ID: 5TZ1), and *C*. *tropicalis* (PDB ID: 5TZ1).

The preparation of protein for docking simulation data involved the removal of all cocrystallized ligands, incorporated water molecules, and nonessential heavy atoms followed by the addition of polar hydrogen and assignment of Gasteiger charges [33–35]. The molecular geometry of both limonene and thymol was optimized using energy minimization before docking simulations. Docking simulations were performed using AutoDock 4.2.6, whereas protein and ligand preparation, grid generation, and docking setup were carried out using AutoDockTools Version 1.5.6 [36, 37]. The AutoDock 4.2.6 system is equipped with the Lamarckian genetic algorithm (LGA) feature, which has been applied for every docking calculation. A summary of the grid box center coordinates and other measurements for all target proteins is shown in Table 1. The procedure of docking was confirmed to be reliable in view of successful redocking of cocrystallized ligands into their own places, giving results with RMSD under 2.0 Å.

**Table 1:** Docking parameters for the selected antimicrobial target proteins, including PDB IDs, cocrystallized ligands, grid box center coordinates, and grid box dimensions.

Complexes	Grid boxes coordinates	Grid center dimensions	Native/cocrystallized ligands
Thym‐2X22	*X* = −21.606	126 × 126 × 126	5‐hexyl‐2‐(2‐methylphenoxy)phenol
	*Y* = −27.755		
Lim‐2X22			
	*Z* = 19.64		
Thym‐3AQC	*X* = 152.385	126 × 126 × 126	(2E,6E)‐7,11 dimethyldodeca2,6,10‐trien‐1‐yl trihydrogen diphosphate
	*Y* = 566.836		
Thym‐3AQC			
	*Z* = 64.303		
Thym‐3G75	*X* = 42.759	126 × 126 × 126	4‐methyl‐5‐[3‐(methylsulfanyl)‐1H‐pyrazol‐5‐yl]‐2‐thiophen‐2‐yl‐1,3‐thiazole
	*Y* = 1.661		
Thym‐3G75			
	*Z* = 21.896		
Thym‐3IX3	*X* = 12.116	126 × 126 × 126	Autoinducer N‐3‐oxo‐dodecanoyl‐L‐homoserine lactone
	*Y* = 1.179		
Thym‐3IX3			
	*Z* = 16.509		
Thym‐5TZ1	*X* = 62.614	126 × 126 × 126	(R)‐2‐(2,4‐difluorophenyl)‐1,1‐difluoro‐3‐(1H‐tetrazol‐1‐yl)‐1‐(5‐(4‐(2,2,2‐trifluoroethoxy)phenyl)pyridin‐2‐yl)propan‐2‐ol
	*Y* = 66.604		
Thym‐5TZ1			
	*Z* = 2.766		
Thym‐5ZXM	*X* = −11.741	126 × 126 × 126	Adenosine‐5 ^′^‐diphosphate (ADP)
	*Y* = −10.774		
Thym‐5ZXM			
	*Z* = 33.492		
Thym‐6F86	*X* = 67.315	126 × 126 × 126	4‐(4‐bromanylpyrazol‐1‐yl)‐6‐(ethylcarbamoylamino)‐N‐pyridin‐3‐yl‐pyridine‐3‐carboxamide
	*Y* = 31.922		
Thym‐6F86			
	*Z* = 54.435		
Thym‐7W9Y	*X* = −23.664	126 × 126 × 126	Nicotinamide adenine dinucleotide phosphate
	*Y* = 34.972		
Thym‐7W9Y			
	*Z* = 0.121		

The molecular geometry of both limonene and thymol was optimized using energy minimization prior to docking simulations. Docking simulations were performed by independently docking limonene and thymol into the active site of each target protein using the crystallographic ligand′s position defining the binding site and reported catalytic residues [38]. Multiple binding conformations were generated and the binding conformation with the lowest binding free‐energy (kcal/mol) and the best ligand/protein interactions were selected as the most favorable conformation [28, 29, 34, 39]. The resulting molecular complexes were analyzed for the hydrogen‐bonding interactions, hydrophobic contacts, van der Waals interactions, *π*‐*π* interactions, and other noncovalent forces stabilizing the complexes. Interaction analyses and visualization were performed using Discovery Studio Visualizer as well as PyMOL software.

## 5. Statistical Analysis

All experiments were performed in triplicate, and data are expressed as mean ± SD. Statistical analysis was performed using a one‐way analysis of variance (ANOVA) followed by Tukey′s post hoc test to compare the antimicrobial activities of thymol, limonene, and reference antibiotics against the tested microorganisms. Differences were considered statistically significant for *p* < 0.05.

## 6. Results and Discussion

### 6.1. Antimicrobial Activity by Disk Diffusion Method

**Figure 1:**
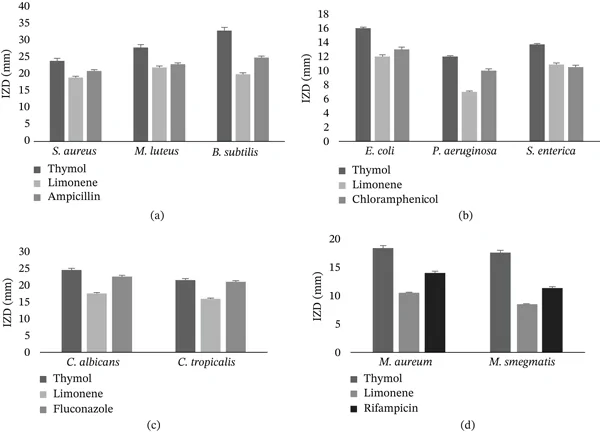
Antimicrobial activity of thymol, limonene, and reference antibiotics against the tested microorganisms. (a) Gram‐positive bacteria, (b) gram‐negative bacteria, (c) yeasts, and (d) mycobacteria. Values are expressed as mean ± SD (*n* = 3). IZD, inhibition zone diameter.

The antimicrobial potential of thymol and limonene was firstly evaluated in vitro via the disk diffusion method against various microorganisms (Table 2 and Figure 1). The disks contained either compound at 100 *μ*g/disk and standard concentrations of AMP (10 *μ*g/disk), CHL (30 *μ*g/disk), FLC (50 *μ*g/disk), and miconazole (10 *μ*g/disk).

**Table 2:** Inhibition zones of thymol, limonene, and antibiotics used against microbial strains.

Microorganism	Type	Thymol (mm)	Limonene (mm)	Antibiotic (mm)	*p* value
*Staphylococcus aureus*	Gram‐positive	24.4 ± 1.5^a^	19.07 ± 1.01^b^	AMP (21.17 ± 0.76)^b^	0.003
*Micrococcus luteus*	Gram‐positive	28.8 ± 1.8^a^	22.17 ± 0.76^b^	AMP (23.07 ± 0.9)^b^	0.001
*Bacillus subtilis*	Gram‐positive	33.1 ± 2.1^a^	20.07 ± 0.9^c^	AMP (25.77 ± 0.68)^b^	< 0.001
*Escherichia coli*	Gram‐negative	16.2 ± 1.1^a^	12.53 ± 0.92^b^	CHL (13.7 ± 0.61)^b^	0.006
*Pseudomonas aeruginosa*	Gram‐negative	12 ± 1.7^a^	7.77 ± 0.81^b^	CHL (10.87 ± 0.81)^ab^	0.016
*Salmonella enterica*	Gram‐negative	12.9 ± 1.7^a^	11.53 ± 0.47^a^	CHL (10.5 ± 0.5)^a^	0.076 ns
*Candida albicans*	Yeast	24.5 ± 0.8^a^	17.47 ± 0.5^c^	FLU (22.5 ± 0.5)^a^	< 0.001
*Candida tropicalis*	Yeast	21.5 ± 1.7^a^	15.87 ± 0.81^b^	FLU (20.97 ± 0.95)^a^	0.003
*Mycobacterium smegmatis*	Mycobacteria	18.4 ± 2.1^a^	10.5 ± 0.5^c^	RIF (14 ± 1)^b^	0.001
*Mycobacterium aureum*	Mycobacteria	17.6 ± 2.3^a^	8.5 ± 0.5^b^	RIF (11.33 ± 0.58)^b^	< 0.001

*Note:* Values are shown as the mean of three repetitions with the standard deviation (mean ± SD). The results were statistically analyzed and compared by using one‐way ANOVA and by performing Tukey′s multiple comparison tests. Different letters found in the same row of either figure will indicate a statistically significant difference between groups (i.e., *p* < 0.05), ns represents nonsignificant results.

Regarding results, the antimicrobial efficacy of thymol surpassed that of limonene. The most sensitive gram‐positive strain was *B*. *subtilis* with an inhibition zone of 33.1 ± 2.1 mm; the subsequent highest were *M*. *luteus* (28.8 ± 1.8 mm) and *S. aureus* (24.4 ± 1.5 mm). Importantly, results indicated that thymol displayed greater antibacterial activity than AMP against the three gram‐positive (*B. subtilis*, *M. luteus*, and *S. aureus*).

Contrary to gram‐positive bacteria, all gram‐negative species tested were generally less susceptible. Indeed, *E*. *coli* was the most sensitive (16.2 ± 1.1 mm) compared with *P. aeruginosa* and *S. enterica* (12 ± 1.7 mm and 12.9 ± 1.7 mm, respectively) when treated with thymol, whereas limonene exerted a less potent effect on gram‐negative bacteria compared with thymol, with inhibition zones ranging from 7.77 to 12.5 mm.

Regarding yeasts and mycobacteria, the disk diffusion results showed that thymol and limonene exerted significant antimicrobial activity against both yeasts and mycobacteria. Importantly, thymol exhibited higher activity than RIF and FLC against *Mycobacterium* and *Candida* species. More specifically, thymol showed strong effect on *C*. *albicans* and *M. smegmatis* (24.5 and 18.4 mm, respectively) compared with the reference antibiotics (22.5 and 14 mm, respectively).

However, since the disk diffusion technique is impacted by the diffusion properties of the compounds, MIC and MBC tests were conducted to quantitatively precise the antibacterial and bactericidal properties of the compounds tested.

**Figure 2:**
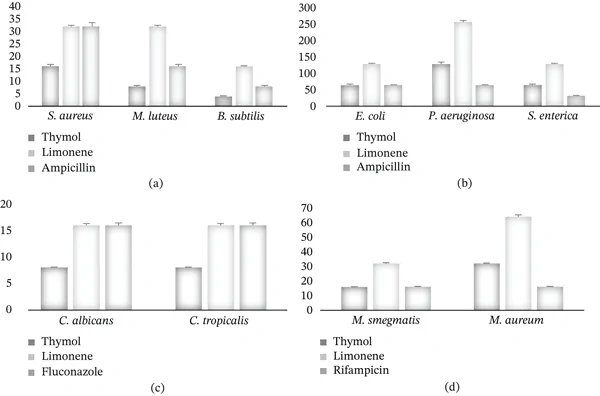
MIC values of thymol, limonene, and reference antibiotics against the tested microorganisms. (a) Gram‐positive bacteria, (b) gram‐negative bacteria, (c) yeasts, and (d) mycobacteria.

Indeed, the microdilution method confirmed the disk diffusion results (Table 3 and Figure 2). The MIC values indicated that thymol consistently exhibited lower MIC values than limonene, demonstrating its considerably stronger antimicrobial activity. Thymol compound also showed more significant effects than reference antibiotics, particularly its efficacy against gram‐positive bacteria (4–16 *μ*g/mL) and against *Candida* species (8 *μ*g/mL), compared with the reference antibiotics, AMP, and FLC, which showed values ranging from 8 to 34 *μ*g/mL.

**Table 3:** MIC values of thymol, limonene, and antibiotic references against bacteria, *Candida*, and *Mycobacterium* species in *μ*g/mL.

Microorganism	Thymol (*μ*g/mL)	Limonene (*μ*g/mL)	Antibiotic	MIC ATB (*μ*g/mL)
*Staphylococcus aureus*	16	32	AMP	32
*Micrococcus luteus*	8	32	AMP	16
*Bacillus subtilis*	4	16	AMP	8
*Escherichia coli*	64	128	CHL	64
*Pseudomonas aeruginosa*	128	256	CHL	64
*Salmonella enterica*	64	128	CHL	32
*Candida albicans*	8	16	FLU	16
*Candida tropicalis*	8	16	FLU	16
*Mycobacterium smegmatis*	16	32	RIF	16
*Mycobacterium aureum*	32	64	RIF	16

More specifically, thymol and limonene both showed a significant effect against the three gram‐positive bacteria, specifically *B*. *subtilis*, which was the most susceptible strain, with MIC values of 4 and 16 *μ*g/mL, respectively.

However, thymol and limonene showed considerably higher MIC values against gram‐negative bacteria, notably *P. aeruginosa*, with MIC values of 128 and 256 *μ*g/mL, respectively, indicating that gram‐negative bacteria are more resistant compared with gram‐positive bacteria.

In addition, thymol and limonene inhibited the growth of *C*. *albicans* and *C*. *tropicalis,* with MIC values of 8 *μ*g/mL for thymol and 16 *μ*g/mL for limonene. The two compounds also showed a moderate effect against *Mycobacterium* species (16–64 *μ*g/mL). Notably, thymol exhibited an effect similar to RIF against *M*. *smegmatis*.

Importantly, the MBC/MIC ratios indicated that both compounds had bactericidal and fungicidal effects against all tested microorganisms, highlighting their potential to kill bacteria and yeasts rather than merely inhibiting their microbial growth (Table 4).

**Table 4:** MBC/MFC values and MBC (MFC)/MIC ratios of thymol and limonene against tested microorganisms.

Microorganism	Thymol MBC/MFC (*μ*g/mL)	MBC (MFC)/MIC	Limonene MBC/MFC (*μ*g/mL)	MBC (MFC)/MIC	Interpretation
*Staphylococcus aureus*	32	2	64	2	Bactericidal
*Micrococcus luteus*	16	2	64	2	Bactericidal
*Bacillus subtilis*	8	2	32	2	Bactericidal
*Escherichia coli*	128	2	256	2	Bactericidal
*Pseudomonas aeruginosa*	256	2	512	2	Bactericidal
*Salmonella enterica*	128	2	256	2	Bactericidal
*Candida albicans*	16	2	32	2	Fungicidal
*Candida tropicalis*	16	2	32	2	Fungicidal
*Mycobacterium smegmatis*	32	2	64	2	Bactericidal
*Mycobacterium aureum*	64	2	128	2	Bactericidal

*Note:* An MBC/MIC or MFC/MIC ratio ≤ 4 indicates bactericidal/fungicidal activity, whereas a ratio > 4 suggests a bacteriostatic/fungistatic effect.

Abbreviations: MBC, minimum bactericidal concentration; MFC, minimum fungicidal concentration; MIC, minimum inhibitory concentration.

The results of the present study are consistent with previous studies showing that plant‐derived molecules have stronger antimicrobial effect against gram‐positive bacteria than gram‐negative bacteria [40], and interesting antifungal activity [41]. However, only a limited number of studies have examined their effect against mycobacteria species [42].

Indeed, the antimicrobial efficacy of thymol aligns with previous reports demonstrating its broad‐spectrum activity against a wide range of microorganisms, including bacteria, yeast, and mycobacteria. Similar results were finding by Cometa et al. [43] who showed the antimicrobial effect of thymol against *S. aureus*, *E*. *coli*, *P. aeruginosa*, and *C*. *albicans* [43].

For instance, Hajibonabi et al. [44] reported that thymol‐encapsulated nanomaterials exhibited enhanced aqueous solubility and improved https://www.sciencedirect.com/topics/pharmacology-toxicology-and-pharmaceutical-science/antibacterial-activity antibacterial properties against *S. aureus*, *S*. *enterica*, *Clostridium perfringens*, and *E*. *coli* compared with free thymol [44].

Moreover, Radochia et al. [45] showed that thymol exhibits potential antimicrobial activity against both bacteria and *Candida* spp., in planktonic and sessile cultures. The MIC and MBC values against bacteria ranged from 125 to 250 *μ*g/mL, whereas the MIC and MFC values against *Candida* spp. ranged from 250 to 2000 *μ*g/mL. Furthermore, thymol showed a synergetic effect with carvacrol at sub‐MIC values in both planktonic and sessile forms. Cytotoxicity assays revealed that thymol was toxic to only two of the tested cell lines, whereas no toxicity was observed in the human urinary cell line [45].

Another interesting study reported that thymol and carvacrol exhibited strong effect on *S. aureus* and *S*. *enterica*, with MIC values ranging from 0.331 to 0.662 mg/mL. Thymol at MIC and 2x MIC achieved substantial inactivation of surface‐adhered *S. aureus* and *S*. *enterica* (± 6.1 log CFU/cm^2^) on stainless steel after short contact times (1 and 10 min) [46].

Thymol has also been reported in the literature to exhibit very strong antifungal properties [47]. De Castro [48] stated that thymol registered significant antifungal effect against *C. albicans* and *Candida krusei*, with MIC values of 78 *μ*g/mL, and against *C. tropicalis* with a MIC value of 39 *μ*g/mL [48].

Regarding its antimicrobial mechanism of action, thymol exerts significant antibacterial effects by affecting the integrity of the cell membrane [9]. Thymol can integrate into the lipid bilayer of cell membranes, thereby altering their physicochemical properties and perturbing membrane fluidity and permeability [49]. Thymol compromises the barrier, leading to leakage of essential intracellular substances such as potassium ions (K^+^) and adenosine triphosphate (ATP). The leakage of K^+^ disrupts osmotic balance, whereas the loss of ATP directly depletes bacterial energy supply, ultimately causing cell death [50]. Notably, the delocalized electron system (double bonds) and hydroxyl group present in both carvacrol and thymol have been identified as the key structural features influencing their activity. These chemical structures enable thymol to act as a proton exchanger, reducing the proton gradient across the cytoplasmic membrane, leading to proton motive force (PMF) collapse and ATP depletion, ultimately resulting in cell death [51]. The antifungal mechanism of action of thymol involves its binding to ergosterol, thereby disrupting its distribution within the fungal cell membrane, weakening its physical strength, and impairing its permeability barrier function. Thymol can also disrupt ergosterol biosynthesis in fungi, thereby compromising the structural integrity of the fungal cell membrane [52].

Antimicrobial potential of limonene is well discussed in several studies against various bacterial species. Limonene is known to be active against several pathogenic bacteria involved in different diseases and in food industry contamination [53]. Indeed, Umagiliyage et al. [54] investigated the antimicrobial activity of limonene liposome against foodborne illness causing bacteria (*E*. *coli* and *Listeria monocytogenes*). The antibacterial activity against *E. coli* showed 0.99 and 1.6 log_10_ reductions in CFU mL^−1^ at 10 *μ*M and 50 *μ*M, respectively, within 48 h. The log_10_ reduction was 1.6 at 10 *μ*M and 3.4 at 50 *μ*M for *L. monocytogenes* [54]. Similarly, Shridar et al. [55] reported that D‐limonene extracted from orange peel inhibited the growth of *E. coli*, *P. aeruginosa*, *S. aureus,* and *C*. *albicans*, whereas it was not effective against *Aspergillus niger* [55]. In a recent report, R‐limonene compound demonstrated a synergistic effect when combined with the *Lippia sidoid*es oil against *S. aureus* strains [56].

Regarding the mechanism of action of limonene, Han et al. [57] showed via scanning electron microscopy (SEM) that limonene caused the destruction of the cell integrity and wall structure of *L. monocytogenes.* The increase in conductivity and the leakage of intracellular biomacromolecules (nucleic acids and proteins) confirmed that limonene had an obvious effect on cell membrane permeability. Furthermore, the decrease in ATP content, ATPase (Na^+^K^+^‐ATPase, Ca^2+^‐ATPase) activity and respiratory chain complex activity showed that limonene could hamper ATP synthesis by blocking the activity of the respiratory complex and ATPase [57]. In another study, Han et al. [58] showed that limonene effectively inhibited bacterial growth at a MIC of 20 ml/L. SEM, the reduction of AKP activity and fluorescence microscope observation confirmed that limonene caused the destruction of the cell morphology and cell wall integrity of *S. aureus*.

Although the absence of an outer membrane in gram‐positive bacteria facilitates the penetration of hydrophobic compounds such as thymol and limonene, other mechanisms also impact the bacterial susceptibility. In gram‐negative bacteria, the outer membrane acts as an additional permeability barrier, whereas multidrug efflux pumps can actively eject antimicrobial products, thereby reducing their intracellular accumulation. Furthermore, differences in membrane lipid composition and membrane fluidity may affect the interaction, partitioning, and disruptive activity of these monoterpenes on the bacterial membrane. Therefore, the antimicrobial action of limonene and thymol is likely governed by multiple structural and physiological factors rather than cell wall architecture alone [59].

Although in vitro studies provide direct evidence of the antimicrobial properties of the tested compounds, they do not fully elucidate the underlying molecular mechanisms. Therefore, computational analysis was performed to gain deeper insights into the possible modes of action, predict the interaction of the studied compounds with potential biological targets, and evaluate their pharmacokinetic and pharmacodynamic characteristics.

## 7. Evaluation of Drug‐Likeness, Pharmacokinetic, and Toxicological Properties of Thymol and Limonene

Thymol and limonene′s pharmacokinetic and drug‐like properties were investigated *in silico* for drug‐like properties to assess their potential to be used as oral bioactive agents. Based on the results shown in Table 5, both of these molecules satisfy all five of Lipinski′s rule of five criteria without any violations, suggesting favorable drug‐like physicochemical properties and the potential for good oral bioavailability. Additionally, both of these compounds exhibit low MW (MWs < 500 Da), moderate lipophilicity (log *P* < 5), and adequate HBD and acceptor capabilities. These physicochemical features are predicted to favor efficient passive diffusion across biological membranes.

**Table 5:** Lipinski′s rules for the thymol and limonene.

	MR	MW	Log *P*	n‐HBA	n‐HBD	Violations Lipinski
**Rule**	**< 140 A** ^**2**^	**< 500 Da**	**≤ 5**	**< 10**	**< 5**	**≤ 2**
**Thymol**	48.01	150.22	2.76	1	1	**Yes**
Limonene	47.12	136.23	3.27	0	0	**Yes**

*Note:* Bold values represent the established threshold criteria of Lipinski′s rule of five used to evaluate the drug‐likeness of the compounds.

In silico ADME and toxicity predictions presented in Table 6 indicate that both compounds are predicted to have high human intestinal absorption (> 93%) and positive BBB permeability values, suggesting their potential ability to reach the central nervous system (CNS). Likewise, the computational models predict that neither thymol nor limonene inhibits the major CYP450 isoforms, indicating a potentially low risk of CYP‐mediated drug–drug interactions. Concerning toxicity, limonene is predicted to be the safer of the two compounds because it tested negative for AMES mutagenicity and hepatotoxicity, whereas thymol tested positive for the risk of hepatotoxicity. However, both compounds are predicted to cause skin sensitization. Overall, these computational predictions suggest that thymol. Overall, this data suggest that thymol and limonene have suitable pharmacokinetics and reasonable safety profiles, providing a solid foundation for more pharmacological research on thymol and limonene.

**Table 6:** ADME and toxicity pharmacokinetic features thymol and limonene.

Models	ADME and toxicity properties													
	Absorption	Distribution		Metabolism							Excretion	Toxicity		
	Intestinal absorption (human)	BBB permeability	CNS permeability	CYP450							Total clearance	AMES toxicity	Hepatotoxicity	Skin sensitization
				Substrate		Inhibitor								
				2D6	3A4	1A2	2C19	2C9	2D6	3A4				
Unity	Numeric (%absorbed)	Numeric (Log BB)	Numeric (Log PS)	Categorical (yes/no)							Numeric (log mL min^−1^ kg^−1^)	Categorical (yes/no)		
Predicted values														
Thymol	93.642	0.57	−1.076	No	No	No	No	No	No	No	0.273	No	Yes	Yes
Limonene	95.898	0.723	−2.37	No	No	No	No	No	No	No	0.213	No	No	Yes

## 8. BOILED‐Egg and Bioavailability Radar Analysis of Thymol and Limonene

**Figure 3:**
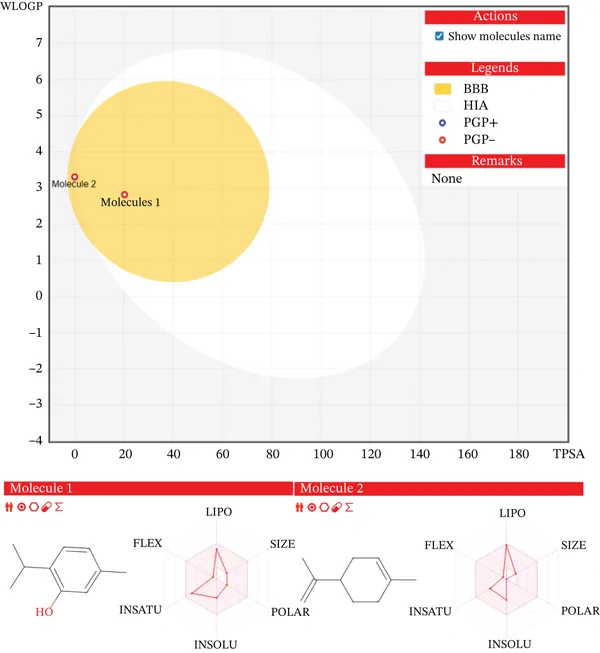
BOILED‐Egg model and bioavailability radar analysis of thymol (Molecule 1) and limonene (Molecule 2).

The oral absorption and access to the CNS of thymol and limonene were predicted using the BOILED‐Egg and bioavailability radar models. As depicted in Figure 3, both compounds occupy the optimal physicochemical region for high gastrointestinal absorption (HIA), suggesting favorable oral bioavailability. In addition, their positions in the BBB region predict a potential ability to penetrate the CNS, which is particularly relevant for compounds targeting the treatment of neurological diseases. Both compounds also exhibit low TPSA and moderate lipophilicity, physicochemical properties that are predicted to favor passive membrane diffusion. Both compounds are also predicted as P‐glycoprotein (PGP−) nonsubstrates, indicating that the likelihood of active efflux from the CNS is low and that they will remain in the CNS for a longer duration. Additionally, the bioavailability radar plots confirm that the six most important physicochemical properties lipophilicity, size, polarity, solubility, flexibility, and saturation are located primarily in the optimal range of values for orally active compounds. These in silico predictions support the potential of thymol and limonene as bioactive molecules with favorable pharmacokinetic features, although experimental validation is required to confirm these findings.

## 9. Molecular Docking Simulations

**Figure 4:**
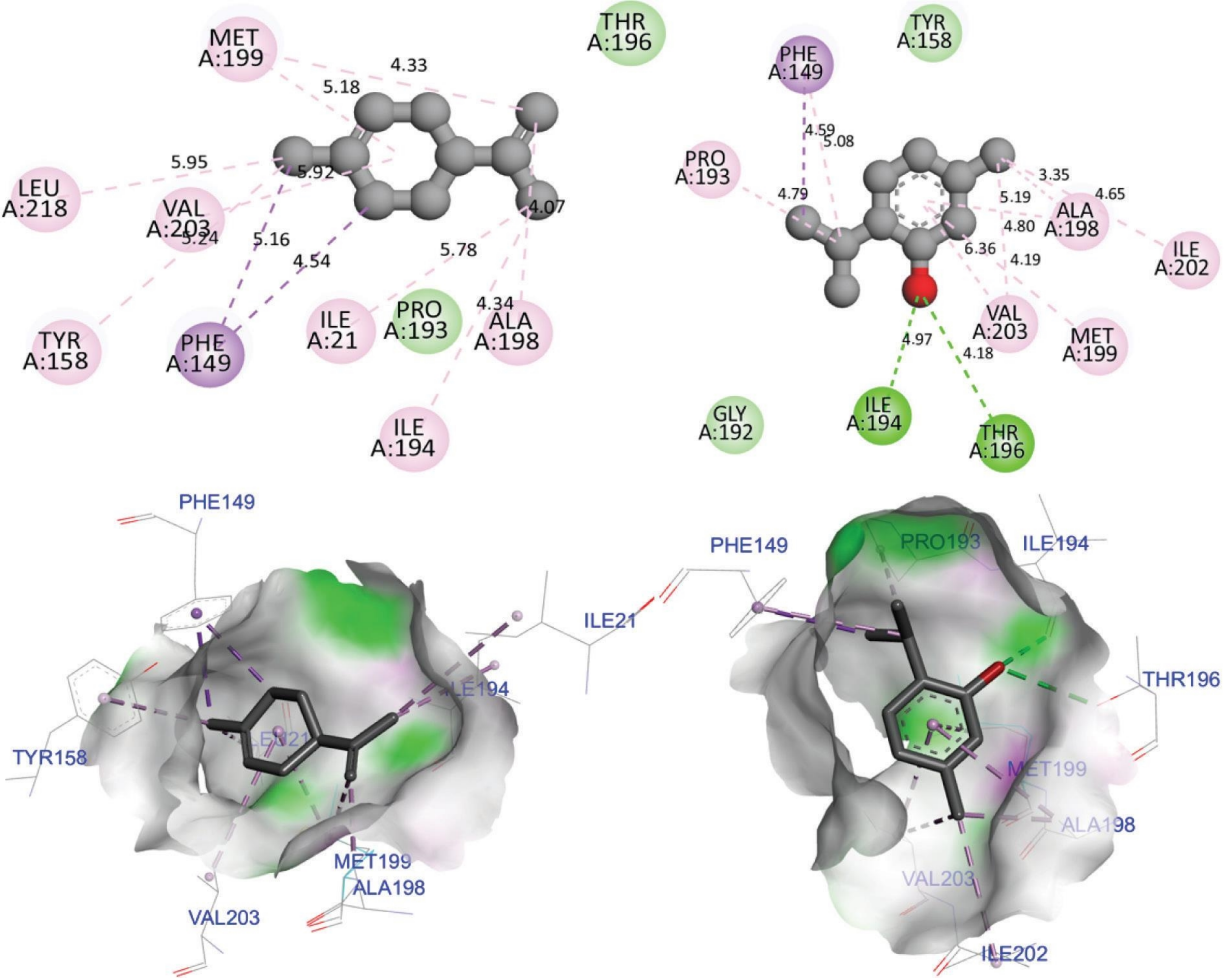
Two‐dimensional and three‐dimensional molecular docking interactions of limonene and thymol within the active site of NADH‐dependent enoyl‐ACP reductase (InhA) from *Mycobacterium tuberculosis* H37Rv (PDB ID: 2X22).

According to the molecular docking analysis shown in Figure 4, both ligands bind to the active site of the NADH‐dependent enoyl‐ACP reductase (InhA) receptor found in *Mycobacterium tuberculosis* (H37Rv) (PDB ID: 2X22), which serves as a structural homolog for both *M*. *aurum* A+ and *M*. *smegmatis* mc^2^155, with nearly equal binding energies of −6.09 kcal/mol (limonene) and −6.11 kcal/mol (thymol). The two compounds bind within the same catalytic cleft, creating similar hydrophobic and *π*‐alkyl interactions with residues VAL A:203, MET A:199, ALA A:198 and PHE A:149; thus providing support for the potential importance of these specific residues in stabilizing these ligands within this essential enzyme for mycobacterial cell wall biosynthesis. Limonene relies entirely on these nonpolar forces for its binding to the receptor, as well as providing contacts with LEU A:218. However, thymol has a more favorable overall energetic profile due to forming strong polar interactions in addition to the shared hydrophobic interactions; through its hydroxyl group, thymol forms hydrogen bonds with both ILE A:194 and THR A:196. This suggests the potential for thymol to offer superior structural stabilization and may interfere with biosynthesis of mycolic acids in the three different strains of *Mycobacterium* evaluated in this study.

**Figure 5:**
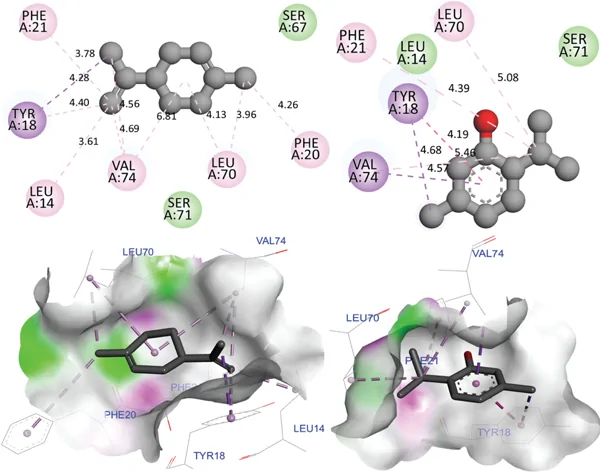
Two‐dimensional and three‐dimensional molecular docking interactions of limonene and thymol within the active site of hexaprenyl diphosphate synthase from *Micrococcus luteus* ATCC 14452 (PDB ID: 3AQC).

As seen in Figure 5, a molecular docking assay has demonstrated the binding of the opposing ligands in the protein, hexaprenyl diphosphate synthase from *M. luteus* ATCC 14452 (PDB ID of 3AGC), where limonene′s binding energy is −5.42 kcal/mol and thymol′s energy is −5.15 kcal/mol. Both compounds are able to bind to the same catalytic pocket and create a common hydrophobic network with PHE A:21, TYR A:18, LEU A:14, VAL A:74, and LEU A:70 via “pi ‐alkyl” and “alkyl” interactions. The forces that limonene uses to interact with these residues are completely from nonpolar interactions and via a specific contact with PHE A:20 to provide maximum structural complementarity; whereas thymol also has the same core hydrophobic footprint but is held in its position via nonpolar interactions (van der Waals forces and pi‐interactions), so that its polar phenolic ring can be located next to SER A:71 of the protein. Given the substantial similarity in residue overlap, limonene and thymol are predicted to bind to the same highly‐conserved and hydrophobic anchoring site, therefore aiding in stabilizing their respective complexes in this gram‐positive bacterial enzyme.

**Figure 6:**
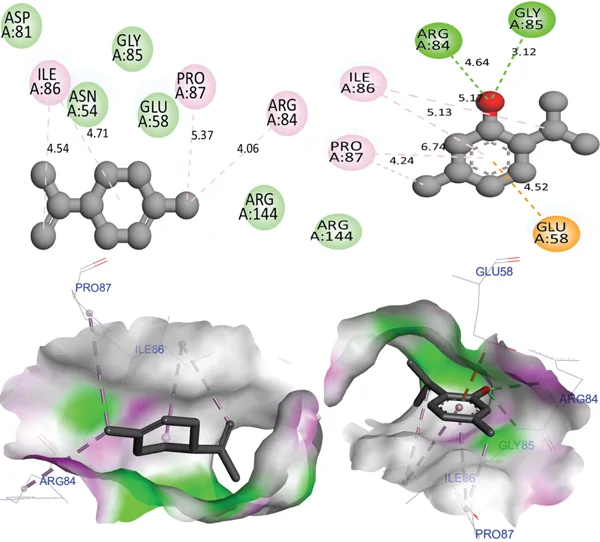
Two‐dimensional and three‐dimensional molecular docking interactions of limonene and thymol within the active site of DNA gyrase subunit B from *Staphylococcus aureus* 017 (PDB ID: 3G75).

The analysis of molecular docking, as depicted in Figure 6, looks at how two ligands bind to the active site of the DNA gyrase B receptor from *S. aureus* 017 (PDB ID of 3G75), with binding energies of −4.92 kcal/mole for limonene and −4.77 kcal/mole for thymol. Additionally, both limonene and thymol interact at a common hydrophobic pocket where both ligands are anchored via hydrophobic alkyl and pi‐alkyl interactions with PRO A:87 and ILE A:86. Limonene binds exclusively through the use of nonpolar interactions within the shared pocket while extending its contact network through specific van der Waals interactions (such as the ones with ASN A:54, GLU A:58, and ARG A:84). On the other hand, thymol uses the same hydrophobic pocket but also has extensive polar interactions due to the presence of its phenolic hydroxyl group, which forms two conventional hydrogen bonds with ARG A:84 and GLY A:85, as well as having an aromatic ring that participates in an electrostatic pi‐anion type of interaction with GLU A:58. This complete overlap suggests that although both phytochemicals utilize the same essential pocket residues, thymol provides a specific mechanism of action for disrupting DNA replication in this gram‐positive clinical isolate due to its ability to form both types of bonds with the corresponding pocket residues in such a way that limonene does not provide.

**Figure 7:**
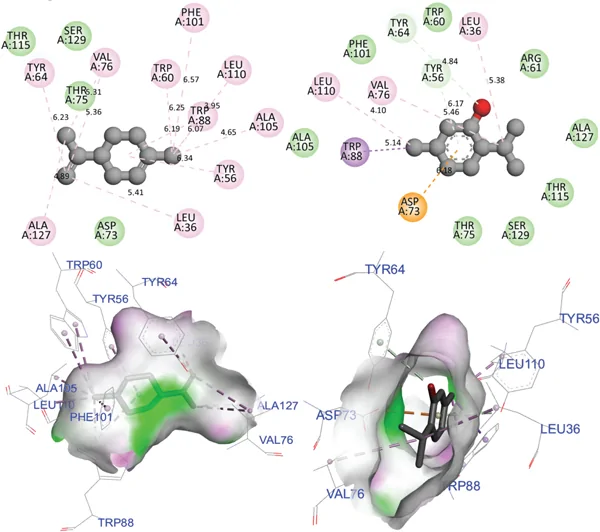
Two‐dimensional and three‐dimensional molecular docking interactions of limonene and thymol within the active site of quorum‐sensing transcriptional regulator LasR from *Pseudomonas aeruginosa* (PDB ID: 3IX3).

Located in the binding cavity of the quorum‐sensing transcription factor (TF) LasR (P01234) from *P*. *aeruginosa* (PDB ID of 3IX3), overviewed in Figure 7, were both bound lauricluorosciutoids and PGC by molecular docking which were measured to have binding energies of −6.19 kcal/mol for limonene and −6.11 kcal/mol for thymol having theoretical differences in binding energy of.07 kcal/mol, based on the close agreement in binding environments as determined through molecular structure analysis. The reported binding environments for each compound reveal considerable overlap of binding sites, consisting of an anchoring network of conserved nonpolar and pi‐alkyl interactions with critical residues (VAL A:76, TRP A:60, TRP A:88, TYR A:56, PHE A:101, LEU A:36, LEU A:110). There are differences between these phytochemicals causing limonene to create a more stable nonpolar anchoring environment entirely by means of covalent bonding (e.g., nonpolar—pi) creating nonpolar contacts in the binding site and having a smaller proportion of electrostatic contact points than at both of their nonpolar anchoring points of thymol due to thymol′s 3%–55% electronegative affinity. It follows from their established residue profiles (e.g., overlap of binding)—both limonene and thymol are predicted to interact with the same critical autoinducer‐binding pocket that may modulate quorum‐sensing signaling pathways in the gram‐negative bacteria (GNB).

**Figure 8:**
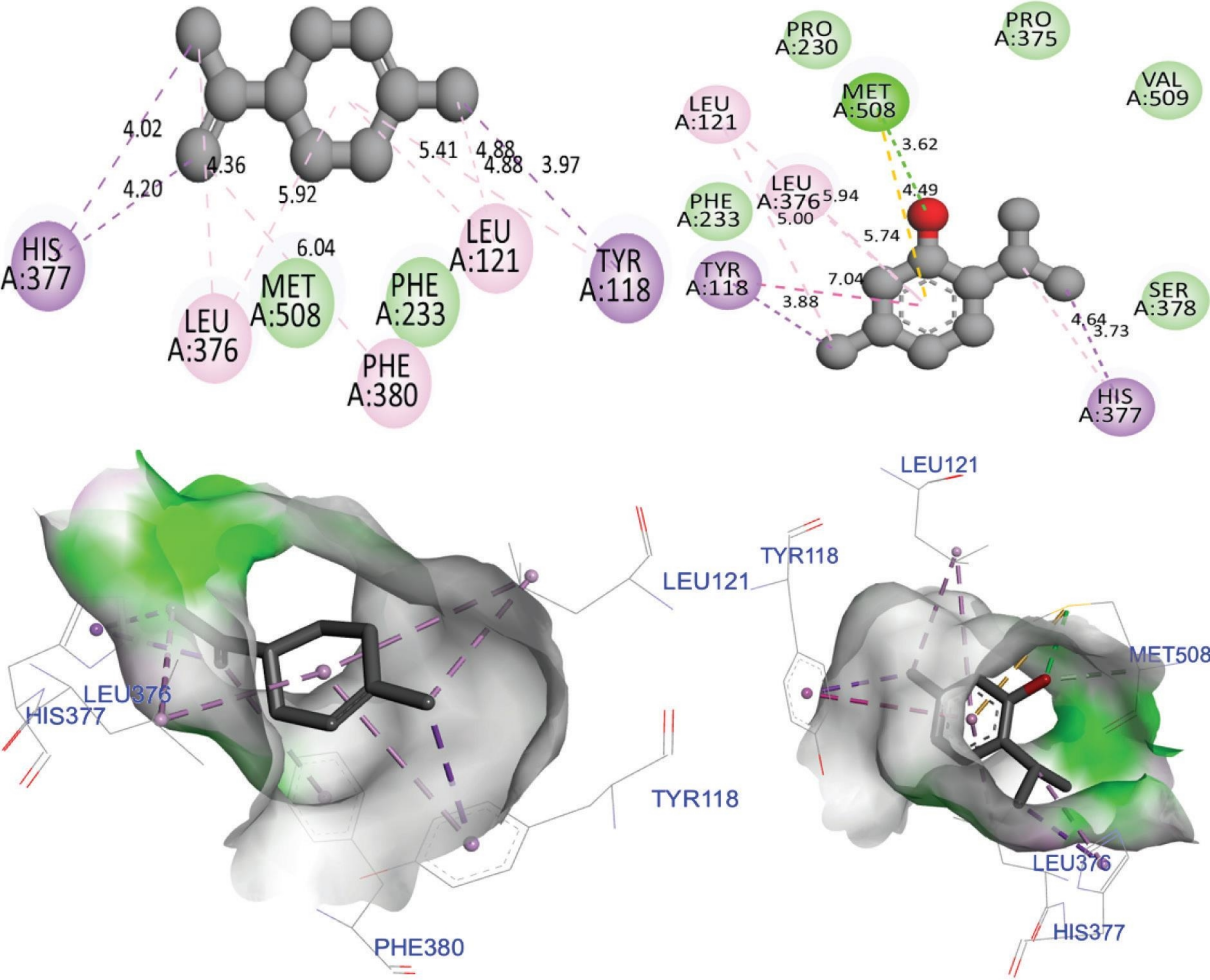
Two‐dimensional and three‐dimensional molecular docking interactions of limonene and thymol within the active site of sterol 14‐alpha‐demethylase (CYP51) from *Candida albicans* (PDB ID: 5TZ1).

The molecular docking analysis, as shown in Figure 8, provides an overview of the binding mechanisms of both antifungal ligands within the active pocket of sterol 14‐alpha‐demethylase (CYP51) from *C*. *albicans* (PDB ID: 5TZ1), which serves as a structural homolog for *C*. *tropicalis* and provides docking energies of −5.87 kcal/mol for limonene and −5.63 kcal/mol for thymol. In addition, Figure 6 illustrates that both antifungal candidates are predicted to bind within the same catalytic pocket, and both have formed a conserved hydrophobic anchoring structure utilizing *π*‐alkyl and alkyl interactions with HIS A:377, LEU A:376, MET A:508, LEU A:121, and TYR A:118. In contrast to limonene which maximizes the contact within this nonpolar area by forming additional contacts with PHE A:380, thymol demonstrated that it shares the same footprint through the formation of a polar interaction via its phenolic hydroxyl group and forming a hydrogen bond network near MET A:508. This similarity in the high density of identical pocket contacts suggests that limonene and thymol may share a similar binding mode, potentially facilitating their interaction with the same conserved pocket residues which are required to destabilize the biosynthetic pathways of fungi entering into ergosterol.

**Figure 9:**
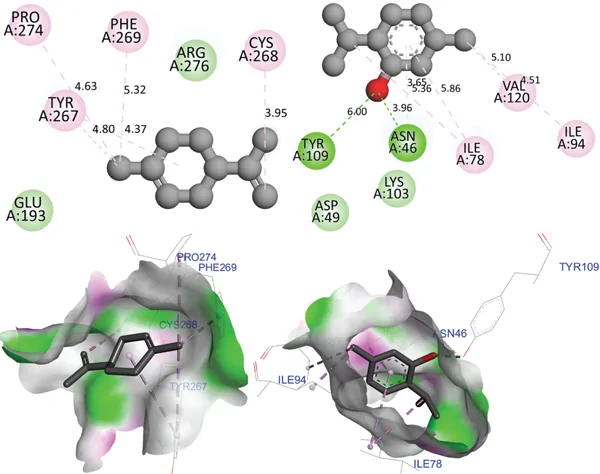
Two‐dimensional and three‐dimensional molecular docking interactions of limonene and thymol within the active site of DNA gyrase subunit B from *Salmonella enterica* (PDB ID: 5ZXM).

The molecular docking analysis illustrated in Figure 9 explores the binding mechanisms of both ligands within the active pocket of the DNA gyrase subunit B receptor from *S*. *enterica* (PDB ID: 5ZXM), yielding binding energies of −5.35 kcal/mol for limonene and −5.54 kcal/mol for thymol. Figure 9 demonstrates that both antimicrobial candidates are predicted to bind within a shared binding cleft, establishing common hydrophobic and pi‐alkyl interactions with crucial residues including TYR A:267, PHE A:269, PRO A:274, and CYS A:268. Limonene acts strictly through these nonpolar forces to adapt its hydrophobic core within the site. In contrast, Thymol expands its shared footprint by incorporating strong electronic stabilization; its phenolic hydroxyl group creates a critical conventional hydrogen bond with TYR A:109 (3.96 Å) and establishes close van der Waals contacts with ASN A:46 and VAL A:120. This highly overlapping configuration underscores that although both phytochemicals exploit an identical structural cleft, thymol′s dual polar and nonpolar binding features anchor it with a more favorable energetic profile against this gram‐negative pathogen.

**Figure 10:**
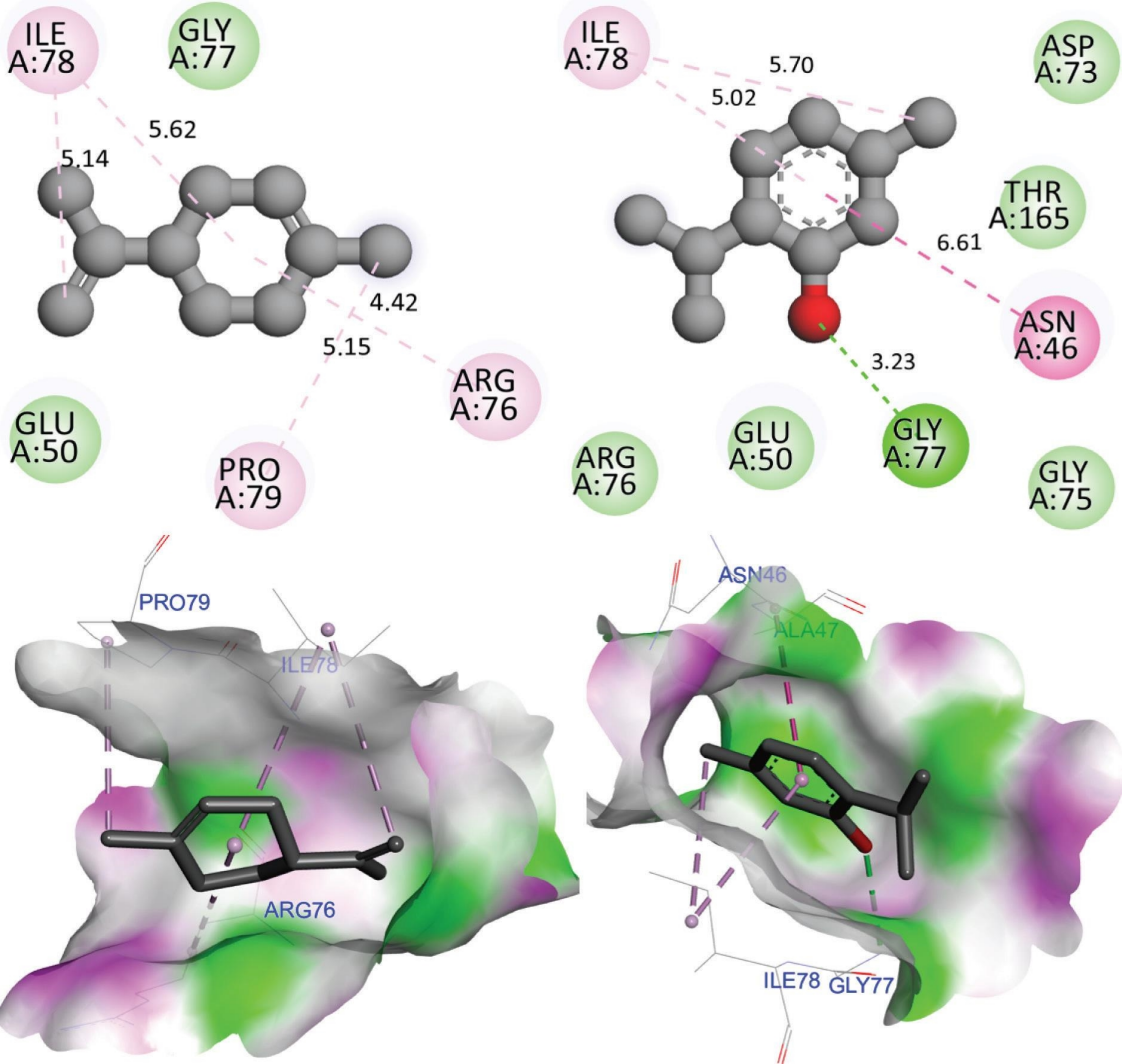
Two‐dimensional and three‐dimensional molecular docking interactions of limonene and thymol within the active site of DNA gyrase subunit B from *Escherichia coli* ATCC 25922 (PDB ID: 6F86).

According to the molecular docking analysis illustrated as Figure 10, the interactions between the respective ligands with the active pocket of the DNA gyrase subunit B receptor from *E*. *coli* ATCC 25922 (PDB ID: 6F86) yield binding energies of −5.05 kcal/mol for limonene and −4.97 kcal/mol for thymol. The analysis of Figure 10 shows that both compounds interact at the same catalytic cleft through shared contacts that are formed by alkyl and pi‐alkyl interactions with ILE A:78 and that both compounds have van der Waals interactions with GLU A:50 that provide close stabilization to this interaction. The interaction of limonene with this nonpolar region is maximized due to the increase in hydrophobic contact it has with PRO A:79 and ARG A:76. The interaction of thymol with this nonpolar region is different in that it utilizes its polar characteristics to adopt its conformation in this shared site through the establishment of a conventional hydrogen bond between the phenolic hydroxyl group of thymol and GLY A:77 (3.23 Å) as well as through establishing further interactions between the core ring of thymol and ASN A:46. The similar structure of both phytochemicals suggests that both compounds may occupy the same essential binding pocket, although the docking analysis suggests the matching binding pocket for Limonene is slightly more optimal based on steric properties for the gram‐negative bacterial target.

**Figure 11:**
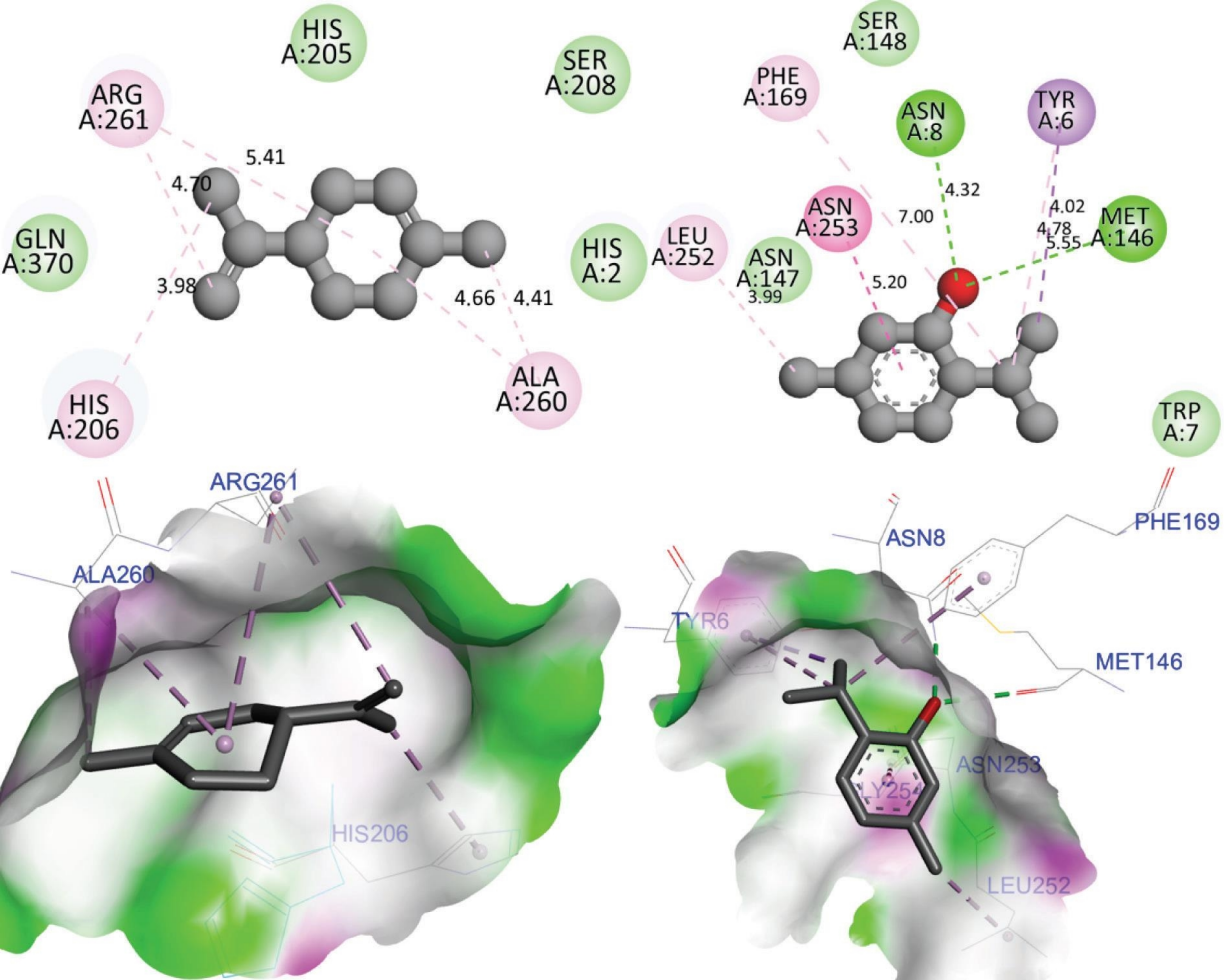
Two‐dimensional and three‐dimensional molecular docking interactions of limonene and thymol within the active site of quorum sensing transcription factor from *Bacillus subtilis* ATCC 6633 (PDB ID: 7W9Y).

In Figure 11, molecular docking analysis reveals the binding mechanisms of both ligands to the active site of the quorum sensing TF, located in *Bacillus subtillis* (PDB ID 7W9Y). The calculated binding energies were −5.62 kcal/mol for limonene and −5.54 kcal/mol for thymol. Figure 11 illustrates that, given their structural differences, both compounds occupy distinct positions within the catalytic binding domain. Limonene is found to lie deep within an apolar pocket and interacts with Arg261, His206, and Ala260 through strong hydrophobic alkyl and pi‐alkyl interactions. In contrast, thymol occupies an adjacent area containing largely aromatic and polar residues and establishes a network of conventional hydrogen bonds with Met146, while also forming a carbon–hydrogen bond with Asn8 via its phenolic hydroxy group. The long axis of thymol′s ring core is anchored by pi‐alkyl interactions with Tyr6 and Leu252. This marked difference in how the two compounds interact with their respective pockets suggests that, although they have similar thermodynamic profiles as they target this gram‐positive bacterium TF, they may interact through nonoverlapping steric and electrostatic pathways.

## 10. Docking Validation Protocol (Redocking)

**Figure 12:**
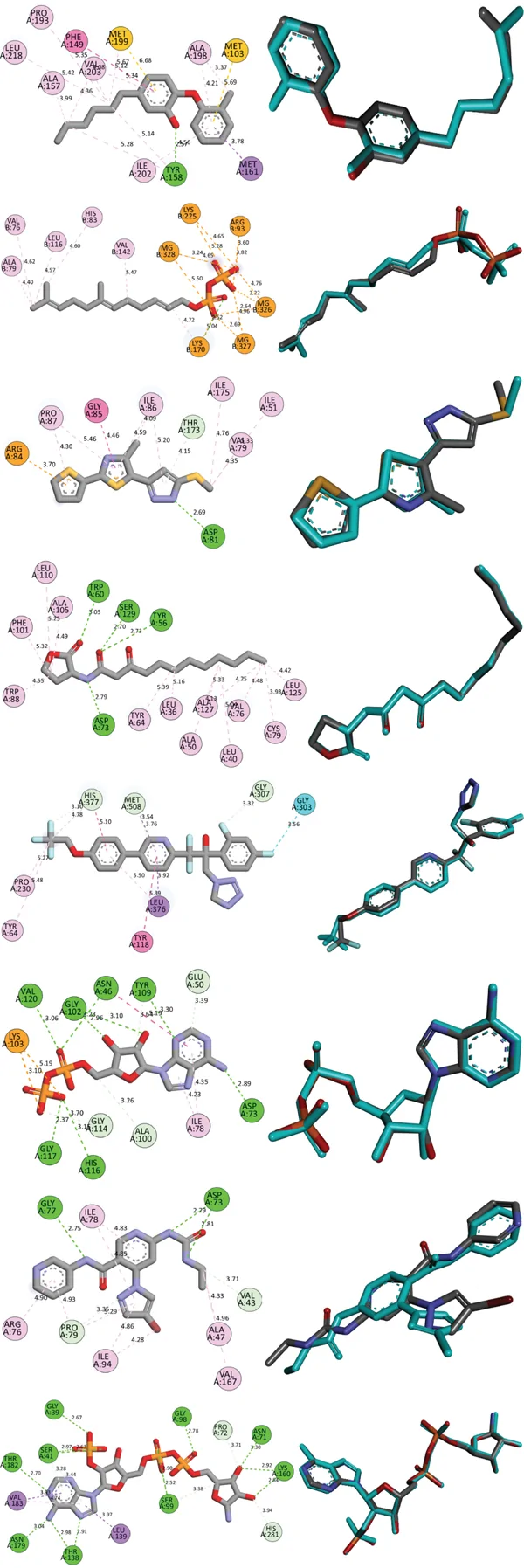
Redocking validation of the cocrystallized (native) ligands in the active sites of PDB structures of 2X22, 3AQC, 3G75, 3IX3, 5TZ1, 5ZXM, 6F86, and 7W9Y, targeting *Mycobacterium tuberculosis*, *Micrococcus luteus* ATCC 14452, *Staphylococcus aureus* 017, *Pseudomonas aeruginosa*, *Candida albicans*, *Salmonella enterica*, *Escherichia coli* ATCC 25922, and *Bacillus subtilis* ATCC 6633, respectively.

To examine the molecular docking protocol, the binding sites of all eight target proteins were used to re‐dock the cocrystallized ligands (Figure 12). The redocking studies showed that all poses reproduced the crystallographic orientations and major intermolecular interactions of the native ligands, thus confirming the validity of the docking parameters and the accurate description of the active‐site cavities. After verification of the docking approach, limonene and thymol were docked into the same experimentally validated binding sites; their binding modes were compared with those of the corresponding native ligands. The superposition of each docked ligand (in cyan) with its respective cocrystallized ligand yielded RMSD values of 0.987, 0.980, 0.994, 0.876, 0.991, 0.787, 1.020, and 0.934 Å, respectively, for 2X22, 3AQC, 3G75, 3IX3, 5TZ1, 5ZXM, 6F86, and 7W9Y, all of which were below the accepted validation threshold of 2.0 Å, thereby confirming the reliability of the docking protocol for subsequent molecular docking analyses [60].

For NADH‐dependent enoyl‐ACP reductase (PDB ID: 2X22), the cocrystallized ligand (5‐hexyl‐2‐(2‐methylphenoxy)phenol) was bound via hydrophobic interactions with Phe149, Pro193, Met199, Ala198, Val203, and Ile202. Limonene and thymol were able to occupy the same binding site and retain the interactions with some of these residues, including Phe149, Pro193, Met199, Ala198, Val203, and Ile202, whereas thymol also retained contacts with Thr196 and Ile194, indicating that the binding mode was similar to that of the natural ligand.

In the case of hexaprenyl diphosphate synthase (PDB ID: 3AQC), the native ligand, namely (2E,6E)‐7,11‐dimethyldodeca‐2,6,10‐trien‐1‐yl trihydrogen diphosphate, showed that important coordination takes place with Mg326, Mg327, and Mg328 and also involves interactions with Lys170, Lys225, and Arg93. However, limonene and thymol do not have a diphosphate moiety to allow the metal‐mediated interactions to occur. Yet it was possible to accommodate both of these compounds in the hydrophobic pocket that has been validated.

For DNA gyrase subunit B having PDB ID of 3G75, its cocrystallized ligand, namely 4‐methyl‐5‐[3‐(methylsulfanyl)‐1H‐pyrazol‐5‐yl]‐2‐thiophen‐2‐yl‐1,3‐thiazole, interacted with residues such as Asp81, Thr173, Gly85, Ile86, Pro87, Arg84, and some other hydrophobic residues. On the other hand, both limonene and thymol maintained interaction with residues Ile86 and Pro87; however, thymol additionally maintained interaction with residues Gly85 and Arg84, therefore being more similar to the native ligand than limonene.

For LasR quorum‐sensing regulator (PDB ID: 3IX3) its cocrystallized ligand, namely autoinducer N‐3‐oxo‐dodecanoyl‐L‐homoserine lactone, was associated with Trp60, Tyr56, Asp73, Ser129, Tyr64, Trp88, Leu36, and Phe101. In the case of limonene, it showed the presence of different hydrophobic contacts with Trp60, Tyr56, Tyr64, Trp88, Phe101, and Leu36; whereas, the case was different for thymol, which additionally maintained contact with Asp73. Regarding sterol 14‐alpha‐demethylase (PDB ID: 5TZ1), the cocrystallized ligand, namely (R)‐2‐(2,4‐difluorophenyl)‐1,1‐difluoro‐3‐(1H‐tetrazol‐1‐yl)‐1‐(5‐(4‐(2,2,2‐trifluoroethoxy)phenyl)pyridin‐2‐yl)propan‐2‐ol, formed a significant bond with His377, Met508, Leu376, Tyr118, Pro230, Gly303, and Gly307. Both limonene and thymol maintained the bond formation with His377, Met508, Leu376, and Tyr118, whereas the former was successful in making a bond with the latter too; however, the bonding with Gly303 and Gly307 did not take place.

For *S*. *enterica* DNA gyrase subunit B (PDB ID: 5ZXM), the crystallized compound, namely adenosine‐5 ^′^‐diphosphate (ADP) made an extensive number of hydrogen bonds with Tyr109, Asn46, Lys103, Asp73, Gly102, Gly114, Gly117, and His116. Thymol preserved its interactions with Tyr109, Asn46, Ile78, and Val120, and limonene did not replicate the main interactions seen in the native ligand, indicating a different binding mode. In the case of *E*. *coli* DNA gyrase subunit B (PDB ID: 6F86), the cocrystallized ligand, namely 4‐(4‐bromanylpyrazol‐1‐yl)‐6‐(ethylcarbamoylamino)‐N‐pyridin‐3‐yl‐pyridine‐3‐carboxamide, interacted mainly with Gly77, Asp73, Ile78, Arg76, and Pro79. Limonene preserved the hydrophobic contacts with Ile78, Arg76, and Pro79, whereas thymol maintained the hydrogen bond with Gly77 along with the interaction with Ile78, indicating that a part of the native interaction network was preserved.

In the case of the quorum‐sensing TF (PDB ID: 7W9Y), the bound ligand NADP (nicotinamide adenine dinucleotide phosphate) formed multiple hydrogen bonds with amino acids Thr182, Ser41, Gly39, Asn179, Thr138, Ser99, Gly98, Asn71, and Lys160 and hydrophobic contacts with Val183 and Leu139. Limonene and thymol did not mimic the observed interactions, as NADP is a big polar cofactor consisting of various phosphates. However, both compounds could occupy the active‐site cavity, making it possible to propose stable conformations based on different interaction patterns.

In general, the accurate reproduction of crystal binding modes of all ligands indicates that the docking method is successfully validated and proves that limonene and thymol bind in the same active sites and keep similar interacting residues for most proteins, indicating that the docking processes are reliable.

## 11. Conclusion

This investigation shows the remarkable antimicrobial potential of thymol and limonene (purity ≥ 98%). These monoterpenoid compounds were found to have significant antimicrobial properties against a wide variety of microorganisms including gram‐positive and gram‐negative bacteria, yeast, and mycobacteria. Thymol generally exhibited greater antimicrobial activity than limonene and reference antibiotics explained by larger zones of inhibition and lower MBC and MIC values. Additionally, the MBC/MIC ratios of both compounds showed that they mostly exert bactericidal or fungicidal actions on all tested strains. These antimicrobial properties of these monoterpenoids may be attributed to their phenolic structure causing disruption of microbial membranes by interfering with basic cellular functions. The ADMET data and molecular docking analyses supported these findings by indicating favorable pharmacokinetic properties, acceptable safety profiles, and stable interactions of both compounds with key bacterial targets. These findings open promising perspectives for the development of synergistic formulations combining thymol and limonene for pharmacological and industrial applications. Future directions should focus on highlighting their mechanisms of action, testing their efficacy against a broader range of clinical and multidrug‐resistant strains, and investigating additional pharmacological and biological effects. Furthermore, in vivo and clinical investigations are required to assess their safety, potential side effects, and therapeutic efficacy before clinical application.

## Author Contributions

**Nesrine Benkhaira**: conceptualization, methodology, software, formal analysis, investigation, data curation, writing—original draft preparation, and writing—review and editing; **Mohamed El Fadili**: methodology, software, formal analysis, investigation, data curation, writing—original draft preparation, and writing—review and editing; **Kawtar Fikri-Benbrahim**: supervision, validation, investigation, writing—review and editing, and visualization; **Saad Ibnsouda Koraichi**: supervision, validation, investigation, writing—review and editing, and visualization.

## Funding

No funding was received for this manuscript.

## Disclosure

All authors have read and agreed to the published version of the manuscript.

## Conflicts of Interest

The authors declare no conflicts of interest.

## Data Availability

The data that support the findings of this study are available from the corresponding author upon reasonable request.
